# Developmental and Functional Expression of miRNA-Stability Related Genes in the Nervous System

**DOI:** 10.1371/journal.pone.0056908

**Published:** 2013-05-20

**Authors:** Érica de Sousa, Lais Takata Walter, Guilherme Shigueto Vilar Higa, Otávio Augusto Nocera Casado, Alexandre Hiroaki Kihara

**Affiliations:** 1 Núcleo de Cognição e Sistemas Complexos, Centro de Matemática, Computação e Cognição, Universidade Federal do ABC, Santo André, SP, Brasil; 2 Departamento de Fisiologia e Biofísica, Instituto de Ciências Biomédicas, Universidade de São Paulo, São Paulo, SP, Brasil; CNRS UMR7275, France

## Abstract

In the nervous system, control of gene expression by microRNAs (miRNAs) has been investigated in fundamental processes, such as development and adaptation to ambient demands. The action of these short nucleotide sequences on specific genes depends on intracellular concentration, which in turn reflects the balance of biosynthesis and degradation. Whereas mechanisms underlying miRNA biogenesis has been investigated in recent studies, little is known about miRNA-stability related proteins. We first detected two genes in the retina that have been associated to miRNA stability, XRN2 and PAPD4. These genes are highly expressed during retinal development, however with distinct subcellular localization. We investigated whether these proteins are regulated during specific phases of the cell cycle. Combined analyses of nuclei position in neuroblastic layer and labeling using anti-cyclin D1 revealed that both proteins do not accumulate in S or M phases of the cell cycle, being poorly expressed in progenitor cells. Indeed, XRN2 and PAPD4 were observed mainly after neuronal differentiation, since low expression was also observed in astrocytes, endothelial and microglial cells. XRN2 and PAPD4 are expressed in a wide variety of neurons, including horizontal, amacrine and ganglion cells. To evaluate the functional role of both genes, we carried out experiments addressed to the retinal adaptation in response to different ambient light conditions. PAPD4 is upregulated after 3 and 24 hours of dark- adaptation, revealing that accumulation of this protein is governed by ambient light levels. Indeed, the fast and functional regulation of PAPD4 was not related to changes in gene expression, disclosing that control of protein levels occurs by post-transcriptional mechanisms. Furthermore, we were able to quantify changes in PAPD4 in specific amacrine cells after dark -adaptation, suggesting for circuitry-related roles in visual perception. In summary, in this study we first described the ontogenesis and functional expression of these two miRNA-stability related proteins in the retina.

## Introduction

Control of gene expression by microRNAs (miRNAs) in the nervous system has been investigated in distinct situations, such as in development [1], [2], [3], cell differentiation [4], [5] and adaptation to ambient demands [6], [7], [8]. miRNA comprises a distinct class of 20–24 nucleotide base pair single-stranded noncoding RNA which post-transcriptionally regulates mRNA copy levels and translation efficiency through complementary binding of small stretches of base pairs, typically in the 3′ untranslated region [9], [10].

The action of these short nucleotide sequences on specific genes depends on intracellular concentration [11], which in turn reflects the balance of biosynthesis and degradation. Whereas mechanisms underlying miRNA biogenesis has been investigated in the last years [12], [13], little is known about miRNA-stability and -degradation related proteins [14]. In this regard, recent studies disclosed the involvement of 5′-3′ exoribonuclease 2, also known as XRN2, in miRNA degradation, and GLD-2 cytoplasmic ribonucleotidyltransferase enzyme, an atypical poly(A) polymerase, aka PAPD4, in miRNA stability [15], [16].

Herein, we thoroughly examined the ontogenesis and the presence of these proteins in retinal neurons, progenitor, glial, and endothelial cells. Finally, functional expression of XRN2 and PAPD4 in the retina was assessed after adaptation to different ambient light conditions.

## Methods

### Ethics Statement

Experiments with animals were conducted in accordance with guidelines of the NIH and the Brazilian Scientific Society for Laboratory Animals. Experimental protocol was approved by the Ethics Committee in Animal Experimentation of the Institute of Biomedical Sciences/University of São Paulo (ICB/USP). All animals were housed in a vivarium with free access to food and water throughout the study.

### Animal Procedures

Experiments were carried out with Long Evans rats (*Rattus novergicus*) kept on a 12 h light/dark cycle (light phase 80–100 lx) with lights on at 06∶00 a.m. Embryonic day 19 (E19) and postnatal day 5 (P5), P10, and P60 rats were euthanized with an overdose of ketamine (30 mg/100 g of body weight, i.m., Parke-Davis, Ann Arbor, MI, USA) and xylazine (2 mg/100 g, i.m., West Haven, CT, USA) between 11∶00 and 12∶00 a.m. In dark-adaptation experiments, there were four groups of animals: Control group, which are adult animals from 12 h light/dark cycle, and those that were kept in the dark for 3 hours, 24 hours, or 24 hours followed by 12∶12 light/dark cycle, all euthanized between 11∶00 and 12∶00 a.m. Next, retinas were removed for different methodologies.

### RNA isolation, cDNA synthesis and Real-Time PCR

Retinas were directly homogenized in 1 ml TRIzol reagent (Invitrogen, Carlsbad, CA, USA) and total RNA was extracted following the manufacturer suggested protocol and previously described [17], [18]. In brief, following two chloroform extraction steps, RNA was precipitated with isopropanol and the pellet washed twice in 70% ethanol. After air-drying, RNA was resuspended in DEPC-treated water and the concentration of each sample obtained from *A*_260_ measurements. Residual DNA was removed using DNase I (Amersham, Piscataway, NJ, USA) following manufacturer protocol. Quantitative analysis of gene expression was carried out with a Rotor-Gene 6000 Real-Time Rotary Analyzer (Corbett Robotics Inc., San Francisco, CA) with specific primers for rat XRN2 (forward, 5′- TCGAGGAGGGCGACAGGGAT-3′; reverse, 5′- GGGCGGTGGCAAAGGGTACT-3′), rat PAPD4 (forward, 5′- ACAGGGTTGTCTACGCCGCC-3′; reverse, 5′- CGCGGGCGTGTTAAGTTGGG-3′). cDNA abundance for GAPDH (forward, 5′-TTCAAAAGAGGGCGCACTTC-3′; reverse, 5′-GCGCACTCTGGTTTTTATTTCA-3′) or cyclophilin (forward, 5′- GCGTTTTGGGTCCAGGAATGGC-3′; reverse, 5′- TTGCGAGCAGATGGGGTGGG-3′) were determined as internal controls. For each 20 µl reverse transcription reaction, 4 µg total RNA was mixed with 1 µl oligodT primer (0.5 µg; Invitrogen) and incubated for 10 min at 65°C. After cooling on ice the solution was mixed with 4 µl 5× first strand buffer, 2 µl of 0.1 M DTT, 1 µl of dATP, dTTP, dCTP and dGTP (each 10 mM), and 1 µl SuperScript III reverse transcriptase (200 U; Invitrogen) and incubated for 60 min at 50°C. Reaction was inactivated by heating at 70°C for 15 min. All PCR assays were performed as follows: after initial activation at 50°C for 2 min and 95°C for 10 min, cycling conditions were 95°C, 10 s and 60°C, 1 min. Dissociation curves of PCR products were obtained by heating samples from 60°C to 95°C, in order to evaluate primer specificity.

### PCR Statistical Analysis

Relative quantification of target gene expression was evaluated using the comparative CT method as previously described in detail [19], [20]. In the present study, control refers to P60, in the developmental experiments, or in the case of dark-adaptation experiments, control refers to animals from 12∶12 light/dark cycle. Values were entered into a one-way analysis of variance (ANOVA), followed by pairwise comparisons (Tukey's HSD test), with the significance level set at 5%.

### Western Blotting

As previously described [21], [22], retinas were rapidly dissected, washed with phosphate buffered saline (PBS), and homogenized in RIPA buffer (50 mM Tris, 150 mM NaCl, 0.1% SDS, 0.5% sodium deoxycholate, 1%Triton X-100 and protease inhibitors). Homogenates were centrifuged for 20 min at 14,000 G, 4°C to remove insoluble material. Protein concentration was determined by the BCA method (Thermo Scientific, Rockford, IL, USA, catalog # 23225) and bovine serum albumin was used as the standard, following manufacturer protocol. Proteins in the membrane preparations were separated by sodium dodecyl sulfate-polyacrylamide gel electrophoresis (SDS-PAGE; 10% gel) and transferred to nitrocellulose membranes. Blots were incubated with 5% non-fat milk in TBST buffer for 2 h at room temperature to block nonspecific binding of the antibodies. After rinsed in TBST, blots were incubated overnight with primary antibodies raised against XRN2, PAPD4 and beta-actin diluted in TBST/3% non-fat milk (Table 1). After the primary antibody incubation, blots were rinsed in TBST and incubated with goat anti-rabbit-peroxidase (ECL^TM^ kit; Amersham, Buckinghamshire, England) for 2 h at room temperature. Detection of labeled proteins was achieved by using the enhanced chemiluminescent system (ECL^TM^ kit; Amersham). Measurements of optical densities were performed using ImageJ software (National Institute of Mental Health, Bethesda, Maryland, USA). Optical densities (OD) of the bands were normalized using the value found for the adult. Data from four independent experiments were entered into a one-way ANOVA, using repeated measures design, followed by pairwise comparisons with Tukey's HSD test.

**Table 1:** Primary antibodies used in immunohistochemistry (IHC) and western blot (WB) experiments.

Antibody	Dilution	Manufacturer	Catalog number
Rabbit anti-XRN2	1:400 (IHC)	Abcam	ab72181
	1:2000 (WB)		
Rabbit anti-PAPD4	1:250 (IHC)	Abcam	ab103884
	1:1000 (WB)		
Mouse anti-cyclin D1	1:100 (IHC)	Santa Cruz Biotechnology	sc-20044
Mouse anti-calbindin	1:100 (IHC)	Sigma-Aldrich	C9848
Mouse anti-parvalbumin	1:100 (IHC)	Sigma-Aldrich	P3088
Mouse anti-GFAP	1:200 (IHC)	Sigma-Aldrich	G3893
Mouse anti-eNOS	1:100 (IHC)	BD Biosciences	610297
Mouse anti-OX42	1:200 (IHC)	AbD Serotec	MCA275R
Mouse anti-beta actin	1:20000 (WB)	Sigma-Aldrich	A5316

### Immunohistochemistry

Eyes were dissected out and the retinas were fixed for 30 minutes in 4% paraformaldehyde (PFA) in phosphate buffer 0.1 M pH 7.3 (PB), and cryoprotected in 30% sucrose solution for at least 24 hours at 4°C. After embedding in O.C.T. compound they were cut transversally (12 μm) on a cryostat. Retinal sections were incubated overnight with primary antibodies in a solution containing 5% normal donkey serum and 0.5% Triton–X 100 in PBS at room temperature. All antibodies and specific concentrations used in this study are listed in Table 1.After several washes, retinal sections were incubated with donkey antiserum against rabbit, mouse or goat IgG tagged to Alexa 488 (1∶250–1∶500, Invitrogen) diluted in 3% normal donkey serum containing 0.5% Triton X-100 in PBS for 2 hours at room temperature. For double-labeling experiments, we used secondary antibodies conjugated to Alexa 546 and Alexa 647 (1∶250–1∶500, Invitrogen). Controls for the experiments consisted of the omission of primary antibodies; no staining was observed in these cases. Counter-staining of retinas was achieved using 4′,6-diamidino-2-phenylindole (DAPI), by incubating sections at room temperature for 10 min. After washing, sections were mounted using Vecta Shield (Vector Labs, Burlingame, CA, USA), and analyzed in Nikon TS100F inverted microscope (Nikon Instruments Inc., Melville, NY, USA). Figures were mounted with Adobe Photoshop CS. Manipulation of the images was restricted to brightness and contrast adjustments of the whole image.

### Image Quantification

Image analyses were performed with Image-Pro Plus (Media Cybernetics, Bethesda, MD, USA) and NIS Elements (Nikon Instruments Inc.), as previously described [23]. After channel separation (RGB) of color images, we performed bitmap analysis. *X*–*Y* axis analyses generated numerical appended data file corresponding to pixel values. The bitmap analysis was used to view the pixel values of the active window (or area of interest, AOI) in numeric format, where values correspond to the brightness of the pixels. In some cases, AOI was defined by the labeling of one channel, and analysis was performed in another channel, as for instance, labeling of XRN2 and PAPD4 in the green channel, defined by DAPI labeling in the blue channel. Values were exported to Excel (Microsoft, Redmond, WA, USA) for the appropriate mathematical analyses. Images and charts were prepared using Adobe Photoshop CS2 (Adobe Systems Inc., San Jose, CA, USA).

## Results

### XRN2 and PAPD4 and are highly expressed in the retina and have distinct gene expression profiles during development

**Figure 1:**
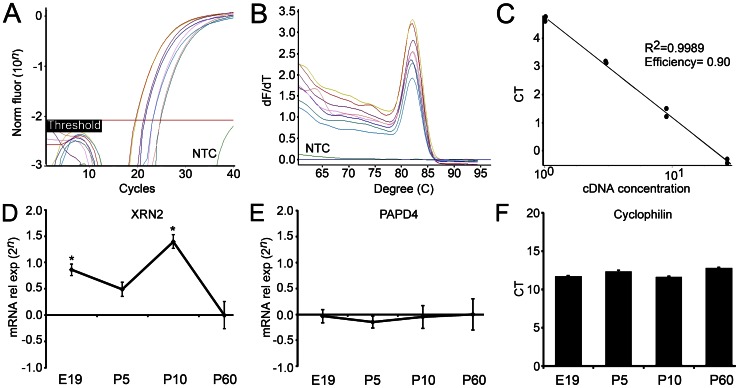
Detection and quantification of XRN2 and PAPD4 gene expression during retinal development. (A) Amplification plots from cDNA serial dilutions (1∶1, 1∶3, 1∶9, and 1∶27) using primers designed for XRN2 gene. Notice that no amplification was obtained in no template control (NTC). (B) Graph of the first derivative plot from normalized fluorescence against temperature (60–95°C). The melting temperature of PCR products (indicated by the peak of the solid lines) matches exactly to the theoretical Tm expected for the amplicon calculated previously. (C) Linear regression analysis using data from different concentrations of cDNA. *R^2^*>0.99 indicates a strong correlation between logarithmic estimated cDNA concentration and cycle threshold (*CT*). Mathematical analysis of linear regression determined the efficiency (*E*) of the primers (1+*E* = 1.90), which are very close to the expected. (D) We detected XRN2 transcripts in retinas from embryonic day 19 (E19), postnatal day 5 (P5) and P10, and the respective levels were compared to P60 (n = 6). Our results revealed that XRN2 has higher expression in retinas from E19 (2?0.86 = 1.82 fold-expression, *P*<0.01) and P10 (2?1.40 = 2.64 fold-expression, *P*<0.01) when compared to P60. (E) We also were able to detect PAPD4 gene expression in all developmental ages. Indeed, our results indicated that PAPD4 transcript levels remain stable from E19 to P60. (F) Gene expression of cyclophilin was used as internal control. Bars represent standard errors of mean. *P<0.01 *vs.* P60 in Tukey's pairwise comparisons after one-way ANOVA.

By using primers specifically designed for XRN2 we generated amplification plots from cDNA serial dilutions. Dissociation curves of these PCR products were obtained by heating samples from 60 to 95°C. The single peak observed matched to theoretical melting temperature calculated previously, indicating specificity of the primers. Linear regression analysis of amplification plots revealed high correlation, confirming amplification linearity (Fig. 1). With the same procedures, we also were able to detect PAPD4 (data not shown).

In addition to the expression in the adult retina, we observed that XRN2 and PAPD4 transcripts are present throughout all analyzed developmental ages, with distinct gene expression profiles (n = 6). Our results indicated that XRN2 has higher expression in E19 retinas (2?0.86 = 1.82 fold-expression, *P*<0.01) and P10 (2?1.40 = 2.64 fold-expression, *P*<0.01) when compared to P60. Otherwise, our results indicated that PAPD4 transcript levels remain stable throughout all studied developmental ages. In these PCR experiments, cyclophilin gene expression was used as internal control (Fig. 1).

### XRN2 and PAPD4 have distinct protein levels during retinal development

**Figure 2:**
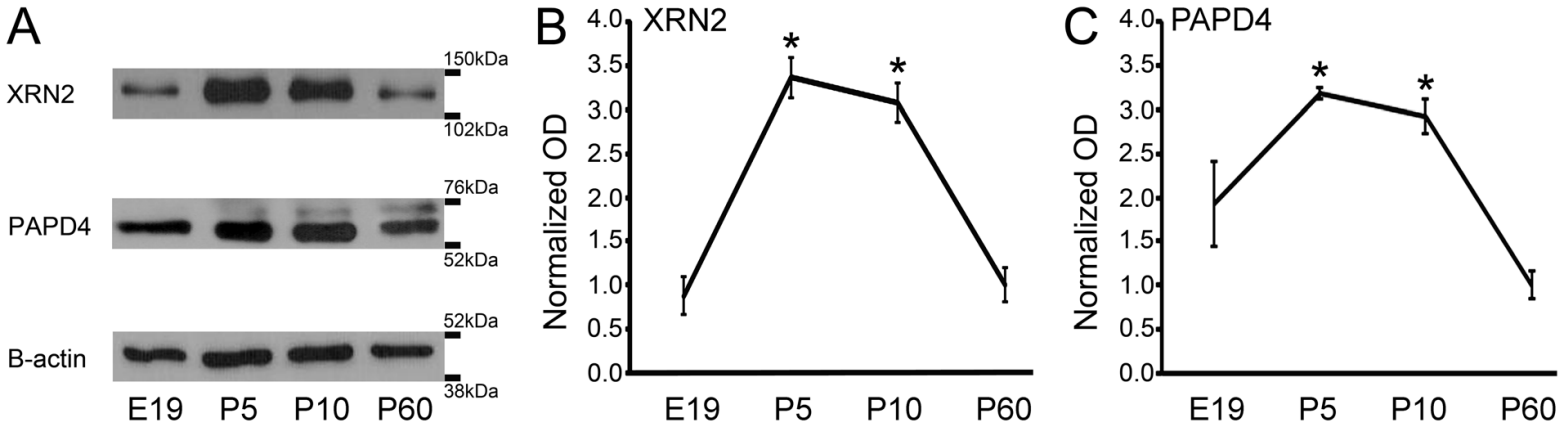
Detection and quantification of XRN2 and PAPD4 protein levels during retinal development. (A) Western blots of XRN2 and PAPD4 in developing and mature retinas. Beta-actin (42 kDa) was used as an internal control. Optical density (OD) from embryonic day 19 (E19), postnatal day 5 (P5) and P10 rats were normalized by OD from adult retinas (P60), in four independent experiments (n = 4). (B) We detected XRN2 in retinas from embryonic day 19 (E19), postnatal day 5 (P5) and P10, and the respective levels were compared to P60.We detected higher levels of XRN2 at P5 (336%, *P*<0.01) and P10 (308%, *P*<0.01). (C) We observed that PAPD4 has a similar developmental profile regarding protein levels. PAPD4 was detected at E19 with similar levels as those found in the adult, however higher levels were detected in P5 (318%, *P*<0.01) and P10 (292%, *P*<0.01) retinas. *P<0.01 *vs.* P60 in Tukey's pairwise comparisons after one-way ANOVA.

Since we were able to detect XRN2 and PAPD4 transcripts in developing retinas, we next examined whether these genes are translated into proteins. In fact, we were able to detect those proteins, although they are specifically regulated during retinal development (n = 4). As shown in Figure 2, we observed that XRN2 is present in E19 retinas with similar levels as P60. However, when compared to P60 and using this age as a reference, we detected higher levels at P5 (336%, *P*<0.01) and P10 (308%, *P*<0.01). PAPD4 has a similar developmental profile regarding protein levels. PAPD4 was detected at E19 with similar levels as those found in the adult, however higher levels were detected in P5 (318%, *P*<0.01) and P10 (292%, *P*<0.01) retinas (Fig. 2).

### XRN2and PAPD4 have similar distribution pattern in developing and mature retina, with distinct subcellular localization

**Figure 3:**
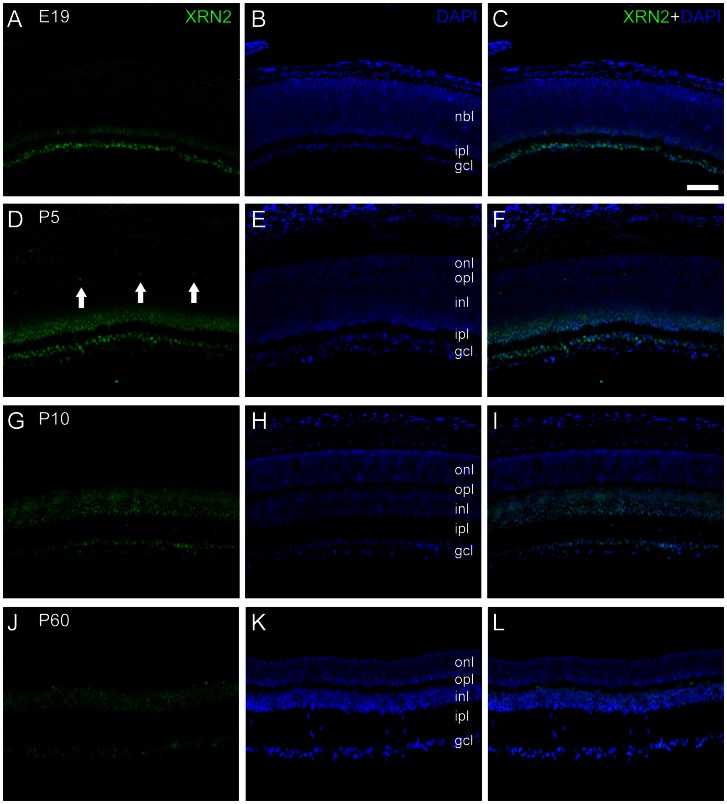
XRN2 immunolabeling in transverse sections of developing and adult rat retinas. XRN2 (green) immunolabeling in sections of developing and adult rat retinas counter-stained with 4′,6-diamidino-2-phenylindole (DAPI, blue). (A–C) In retinas of 19-day embryos (E19) XRN2 immunoreactivity was observed in nuclei located in the ganglion cell layer and in the inner border of the neuroblastic layer. (D–F) At P5, XRN2 nuclear labeling was observed in the ganglion cell layer, and in the inner part of the inner plexiform layer. In addition, some nuclei were seen in the outer border of the inner nuclear layer (arrows). (G–I) In retinas from P10 animals, XRN2 immunoreactivity was observed in ganglion cell layer, and in the entire inner nuclear layer, as well. (J–L) In P60, spatial pattern was very similar to that observed in P10. Labels indicate the approximate location of the neuroblastic layer (nbl), outer nuclear layer (onl), outer plexiform layer (opl), inner nuclear layer (inl), inner plexiform layer (ipl), and ganglion cell layer (gcl). Scale bar: 60 µm.

Employing immunohistochemistry, we next examined XRN2 and PAPD4 spatial distribution in developing and adult retina. For these descriptions, 4–5 retinal sections were analyzed (n = 3). XRN2 typical labeling was mostly observed in cell nuclei, but we also detected a fine punctate in plexiform and nuclear layers, which could be detected with high magnification. In E19 retinas, XRN2 labeling was observed in nuclei located in the ganglion cell layer, and also in the inner border of the neuroblastic layer (Fig. 3). At P5, labeling was observed in the ganglion cell layer and also in the inner third of the inner nuclear layer. In addition, some nuclei located in the outer border of the inner nuclear layer were also seen. In P10 and P60 retinas, we observed a very similar distribution pattern, with XRN2-positive nuclei located in the ganglion cell layer and throughout the entire inner nuclear layer, although labeling seemed brighter at P10 (Fig. 3).

**Figure 4:**
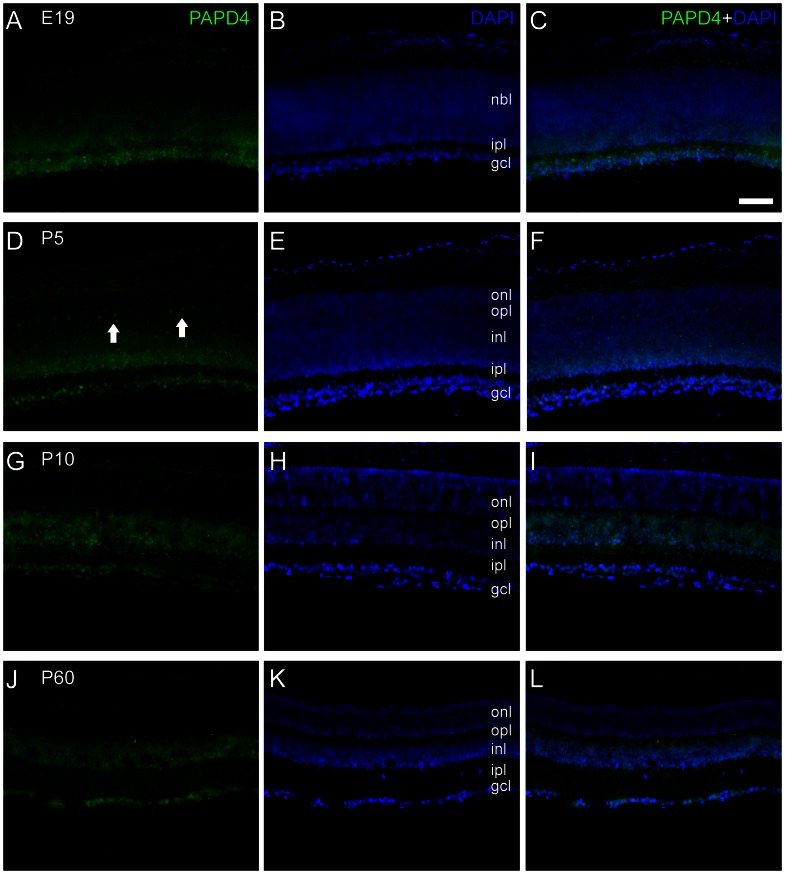
PAPD4 immunolabeling in transverse sections of developing and adult rat retinas. PAPD4 (green) immunolabeling in sections of developing and adult rat retinas counter-stained with 4′,6-diamidino-2-phenylindole (DAPI, blue). (A–C) In retinas of 19-day embryos (E19) PAPD4 immunoreactivity was observed as cytosolic and nuclear labeling in the ganglion cell layer and in the inner part of the neuroblastic layer. Some punctate was also observed in the inner plexiform layer, as well. (D–F) At P5, we observed PAPD4 labeling in the ganglion cell layer, in the inner part of the inner nuclear layer and in some cells located in the outer border of the inner nuclear layer (arrows). In addition, punctate labeling was seen in the inner plexifom layer. (G–I) In P10 retinas, we observed PAPD4 labeling in the ganglion cell layer and in the entire inner nuclear layer, and also punctate labeling was also seen in the inner plexiform layer, as well. (J–L) In P60, spatial pattern was very similar to that observed in P10. Labels indicate the approximate location of the neuroblastic layer (nbl), outer nuclear layer (onl), outer plexiform layer (opl), inner nuclear layer (inl), inner plexiform layer (ipl), and ganglion cell layer (gcl). Scale bar: 60 µm.

In developing and mature retina, we observed that PAPD4 immunolabeling in the retina is often located in perikarya, but we also detected labeling as a fine punctate. This pattern suggests for predominantly cytosolic localization, which is compatible with detection in nuclear and plexiform layers, although nuclear labeling was also observed (Fig. 4). Indeed, in E19 retinas, we observed that PAPD4 is present in the ganglion cell layer. Moreover, we observed a faint labeling in the inner plexiform layer and in the inner part of the neuroblastic layer. At P5, we observed PAPD4-positive cells in the ganglion cell layer and in the inner half of the neuroblastic layer. Moreover, punctate labeling was seen in the inner plexiform layer. At P10 and P60, we observed PAPD4-positive cells located in the ganglion cell layer and in the inner nuclear layer, as well as punctate labeling in the inner and outer plexiform layers (Fig. 4).

### XRN2 and PAPD4 do not accumulate in retinal progenitor cells

**Figure 5:**
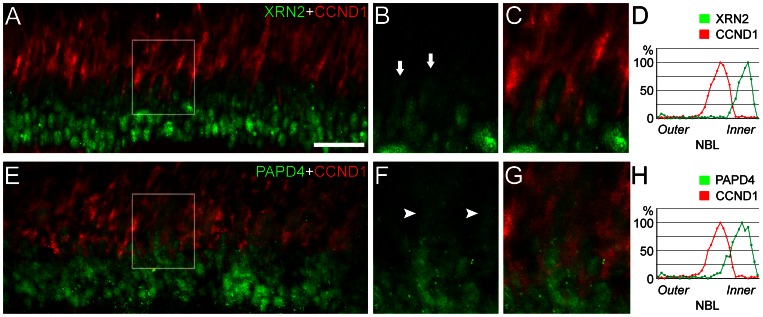
XRN2 and PAPD4 localization in cyclin D1-positive neural progenitor cells. (A) In order to verify the presence of XRN2 (green) in cyclin D1-positive cells (CCND1, red), we performed double-labeling experiments in vertical sections of P5 rat retinas. At this developmental stage, XRN2 was observed at the inner part of the neuroblastic/inner nuclear layer. (B–C) In high magnification of selected areas, it was possible to observe that cyclin D1 did not colocalize with XRN2 (arrows). (D) Pixel analyses indicated that XRN2 and cyclin D1 distribution is quite distinct in the developing retina. (E) We also performed PAPD4 (green) and CCND1 (red) double- labeling experiments. (F–G) In high magnification of selected areas it was possible to detect some degree of colocalization with CCND1 (arrowheads). (H) Pixel analysis of PAPD4 and CCND1 distribution confirm that these proteins in fact share small degree of colocalization in the neuroblatic/inner nuclear layer. Scale bar: 60 µm.

Once we detected the presence of XRN2 and PAPD4 in the developing retina, we examined whether these proteins could be directly involved in developmental processes, such as progression of cell cycle and progenitor cell proliferation. As observed throughout most of the central nervous system (CNS), the nuclei of retinal progenitor cells migrate up and down throughout the depth of the retinal tissue. S-phase occurs at the basal surface, while mitosis occurs at the apical surface, adjacent to the retinal pigmented epithelium [22], [24]. The nuclei migrate between these two surfaces during G1 and G2 in a process called interkinetic nuclear movement [25]. During neurogenesis, the differentiated cells, such as ganglion and amacrine cells, are located in the ganglion cell layer and at the inner part of neuroblastic layer, respectively. We took advantage of this well-known pattern to characterize the presence of XRN2 and PAPD4 in progenitor and post-mitotic neurons. Considering the presence of XRN2- and PAPD4-positive nuclei in the inner part of the developing neuroblastic layer, we ruled out the possibility that these proteins accumulate in mitotic cells. In order to access whether these proteins are present in progenitor cells in S phase, we performed double-labeling experiments with anti-cyclin D1. For this purpose, we analyzed 4–5 retinal sections (n = 3). Although some proximity could be observed between XRN2-positive cells, no colocalization with cyclin D1 was observed in P5 retinas (Fig. 5). Similarly, PAPD4 and cyclin D1labeling showed small degree of overlapping signals in the developing retina (Fig. 5). Altogether, these results revealed that both XRN2 and PAPD4 do not typically accumulate in retinal progenitor cells.

### Horizontal cells accumulate XRN2 and PAPD4 in developing and mature retina

**Figure 6:**
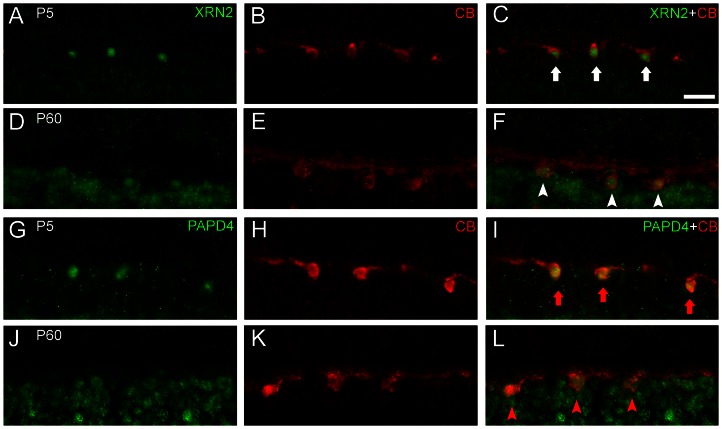
XRN2 and PAPD4 accumulate in horizontal cells in developing and adult retinas. (A–F) To determine whether XRN2 (green) is present in horizontal cells, we performed double-labeling experiments with anti-calbindin (CB, red), a marker for horizontal cells in the rat retina. Indeed, we observed XRN2-positive nuclei in horizontal cells labeled with anti-calbindin, in vertical sections of P5 (white arrows) and P60 (white arrowheads) retinas. (G–L) Similarly, we observed that PAPD4 also accumulates in horizontal cells, in both P5 (red arrows) and P60 rat retinas (red arrowheads). Scale bar: 60 µm.

The mature vertebrate retina is mainly comprised of seven major cell types, namely ganglion, amacrine, horizontal and bipolar cells, cone and rod photoreceptors and glial cells. The generation of the correct proportions of each cell type is crucial for visual function and depends on the appropriate control of proliferation and cell-cycle exit. Since we determined that both proteins do not typically accumulate in retinal progenitor cells, we next examined whether these proteins are present in specific neurons in the developing and adult retina (Fig. 6). Double-labeling experiments using anti-calbindin, a marker for horizontal cells in the rodent retina [26], [27], revealed that both XRN2 and PAPD4 are present in these cells. In P5 retinas, after the completion of horizontal cell differentiation, we observed that positive-calbindin cells accumulate XRN2 and PAPD4. This result was also observed in the adult retina, revealing that the expression of XRN2 and PAPD4 is not transient in differentiated horizontal cells (Fig. 6). For this description, we analyzed 4–5 retinal sections (n = 3).

### XRN2 and PAPD4 expression is mainly neuronal rather than glial in the ganglion cell layer

In spite of differences in developmental expression and subcellular localization, a common feature of XRN2 and PAPD4 was the steady presence in the ganglion cell layer (GCL), in developing to adult retina. This layer comprises diverse cell types, including displaced amacrine cells, several ganglion cell subpopulations and also glial cells. Although distinction of this cell diversity is not easy on pure morphological analysis, astrocytes somata are mainly located in the inner part of the ganglion cell layer. In order to determine whether XRN2 and PAPD4 expression occurs in astrocytes, we performed double-labeling experiments employing anti-GFAP.

**Figure 7:**
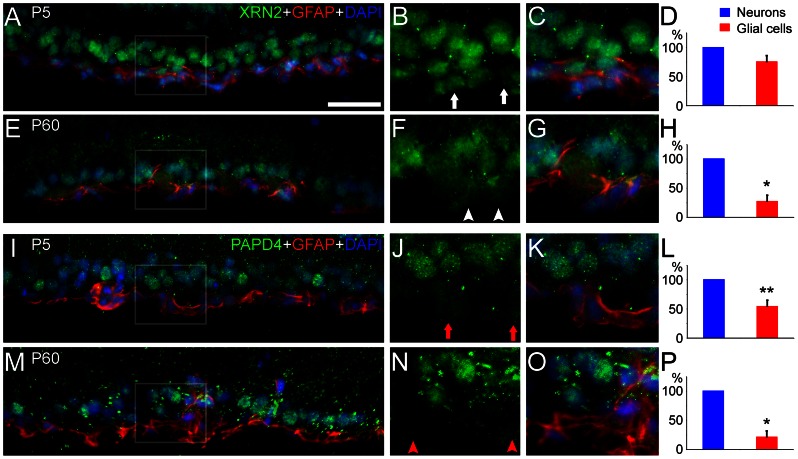
XRN2 and PAPD4 are highly expressed in neurons rather than in astrocytes in the ganglion cell layer. (A, E) In order to verify the expression of XRN2 (green) in glial cells, we performed double-labeling experiments using anti-glial fibrillary acidic protein (GFAP, red) in vertical sections of P5 and P60 retinas counter-stained with 4′,6-diamidino-2-phenylindole (DAPI, blue). Astrocytes are typically located in the inner part of the ganglion cell layer, where GFAP labeling confirmed that these cells are astrocytes. (B, C) In high magnification of selected areas, it is possible to see that XRN2 labeling is weaker in astrocytes nuclei when compared to other nuclei located in the ganglion cell layer in P5 retinas (white arrows). (F, G) Differences between XRN2 labeling in astrocytes and neuronal nuclei are more evident in P60 retinas (white arrowheads). (D, H) Quantification of pixel analysis (n = 3) revealed that XRN2 labeling in astrocytes nuclei is weaker (28%, P<0.01) when compared to that found in neuronal nuclei in P60. Median values found in neuronal nuclei were used to normalize fluorescence levels within the respective image. (I, M) We also examined PAPD4 distribution in vertical sections of P5 and P60 retinas. (J, K) In high magnification of selected areas, we observed that PAPD4 labeling is weaker in astrocytes nuclei in P5 retinas (red arrows). (N, O) Differences between PAPD4 labeling in astrocytes and neuronal nuclei are prominent in P60 retinas (red arrowheads). (L, P) Indeed, quantification of pixel analysis (n = 3) revealed that PAPD4 labeling in astrocytes nuclei is weaker when compared to that found in neuronal nuclei located in the ganglion cell layer, both in P5 (55%, P<0.05) and P60 retinas (22%, P<0.01). *P<0.01 and **P<0.05 in paired T-Test. Scale bar: 60 µm.

Indeed, it is possible to see that labeling of both XRN2 and PAPD4 are brighter in the outer half of the GCL, suggesting that these proteins accumulate in neurons rather than in astrocytes (Fig. 7). Accordingly, nuclei located in the inner part of the ganglion cell layer are often surrounded by GFAP labeling, confirming that these nuclei in fact are from astrocytes. We next compared immunolabeling of XRN2 and PAPD4 in astrocyte and neuronal nuclei. Quantification from pixel analyses (n = 3) confirmed that XRN2 labeling in neuronal nuclei is brighter when compared to that observed in astrocytes nuclei (28%, *P*<0.01) in P60 retinas. In turn, values from PAPD4 labeling in neuronal nuclei is higher when compared to those from astrocytes, in P5 (55%, *P*<0.05) and P60 (22%, *P*<0.01) retinas. Altogether, these results revealed that XRN2 and PAPD4 expression is mainly neuronal rather than glial in the GCL.

**Figure 8:**
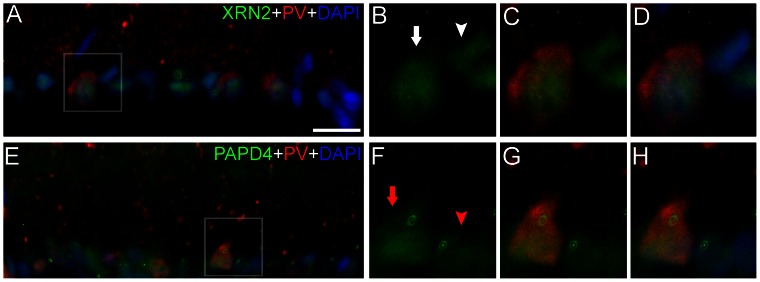
XRN2 and PAPD4 accumulate in α-ganglion cells in the ganglion cell layer. (A) To investigate the presence of XRN2 (green) in specific neurons in ganglion cell layer (GCL), we performed double-labeling experiments using anti-parvalbumin (red), a marker for α-ganglion cells located in GCL, in vertical sections of rat adult retinas counter-stained with 4′,6-diamidino-2-phenylindole (DAPI, blue). (B–D) In high magnification of selected areas, we were able to detect the presence of XRN2 in parvalbumin-positive cells (white arrow), and in other cells located in the GCL, as well (white arrowhead). (E) In addition, we also examined the presence of PAPD4 in these cells. (F–H) Likewise, in high magnification of selected areas, we observed the presence of PAPD4 in parvalbumin-positive cells (red arrow), as well as in other cells located nearby in the GCL (red arrowhead). Scale bar: 60 µm.

Considering that the presence of amacrine cells in the GCL is well established [28], we further investigate the presence of XRN2 and PAPD4 in specific neurons located in this layer. Indeed, there are several markers for ganglion cells described in the literature [29]. Herein, we performed double-labeling experiments with parvalbumin, which is a reliable marker for α-ganglion cells in the rodent retina [26]. We were able to detect both XRN2 and PAPD4 in parvalbumin-positive cells located in the GCL, revealing the presence of these proteins in α-ganglion cells (Fig. 8). For this description, we analyzed 4–5 retinal sections (n = 3).

### XRN2and PAPD4 were not detected in retinal endothelial and microglial cells

**Figure 9:**
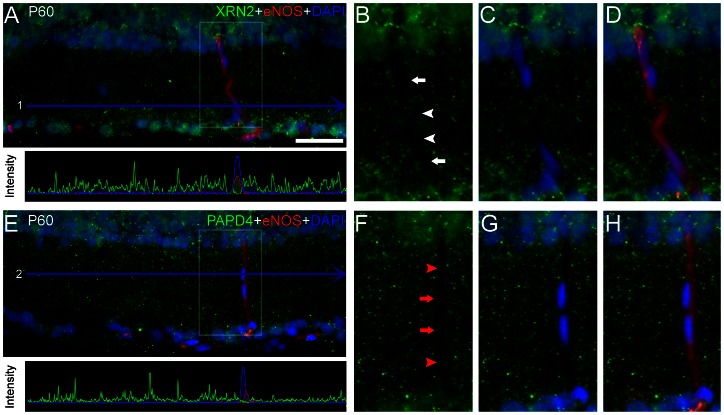
XRN2 and PAPD4 were not detected in endothelial nitric oxide synthase (eNOS)-positive cells. (A, upper ) In adult retinas it is possible to identify endothelial cells in the plexiform layers, especially in the inner plexiform layer. We investigated whether XRN2 (green) accumulate in endothelial cells performing double-labeling experiments using anti-eNOS (red) in retinal sections counter-stained with 4′,6-diamidino-2-phenylindole (DAPI, blue). (A, lower) Quantification of RGB channels pixels intensity within the blue line 1. Notice that green signal inside the red- and blue-delimited areas is virtually absent. (B–D) In high magnification of selected areas, we observed that elongated nuclei from endothelial cells do not accumulate XRN2 (white arrows). In fact, XRN2 labeling was not detected in vessels running vertically in the inner plexiform layer (white arrowheads). (E, upper) Double-labeling experiments were carried out to identify colocalization between PAPD4 (green) and endothelial cells (red) in retinal sections counter-stained with DAPI (blue). (E, lower) Quantification of RGB channels pixels intensity within the blue line 2. Notice that green signal inside the red- and blue-delimited areas is virtually absent. (F–H) In high magnification of selected areas, we observed that PAPD4 do not accumulate in endothelial cells nuclei (red arrows) and cytosol (red arrowheads). Scale bar: 60 µm.

Once we determined that XRN2 and PAPD4 accumulate in differentiated rather than in progenitor cells, and also that the expression occurs mainly in neurons than in astrocytes, we next examined whether these proteins are present in endothelial cells. For this purpose, we performed double- labeling experiments using anti-endothelial NOS (eNOS), a marker for this cell type. In transverse sections of adult retina, anti-eNOS labels vessels crossing vertically the inner plexifom layer, where typical elongated nuclei were observed (Fig. 9). In our experiments, we were not able to detect XRN2 labeling in endothelial cell nuclei defined by DAPI, neither in endothelium cytoplasm. Similar results were obtained for PAPD4, since we also failed to detect accumulation of this protein in endothelial cells (Fig. 9). For this analysis, we observed 4–5 retinal sections (n = 3).

**Figure 10:**
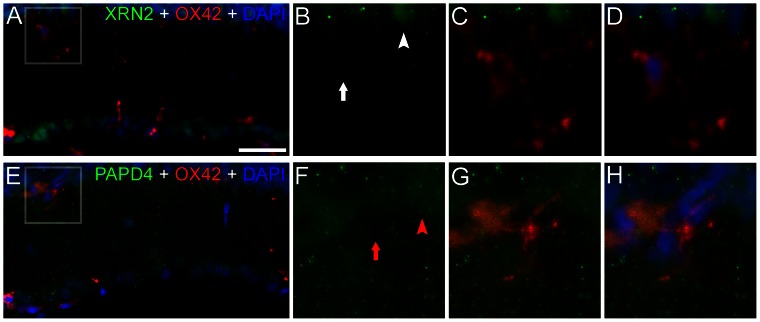
XRN2 and PAPD4 do not accumulate in microglial cells. (A) In order to verify the presence of XRN2 (green) in microglial cells, we employed anti-OX42 (red) in double labeling experiments performed in vertical sections of rat adult retinas counter-stained with 4′,6-diamidino-2-phenylindole (DAPI, blue). (B–D) In high magnification of selected areas, we identified the nuclei of microglial cells, which were not stained for XRN2 (white arrow), in spite of the positive labeling in surrounding areas (white arrowhead). (E) We also examined the presence of PAPD4 in microglial cells. (F–H) In high magnification of selected areas, we were able to identify nuclei of microglial cells, which were not positive for PAPD4 (red arrow), although the presence of this protein was detected in cells located nearby (red arrowhead). Scale bar: 60 µm.

In addition, we also examined the presence of XRN2 and PAPD4 in microglial cells. In this study, we employed anti-OX42, a well-known marker for microglial cells in the CNS, including the retina, in both activated and inactivated states [30]. Indeed, we were not able to detect XRN2 and PAPD4 labeling in the OX42-positive cells (Fig. 10). These results indicated that these proteins are virtually absent in microglial cells. For this description, we analyzed 4–5 retinal sections (n = 3).

### PAPD4, but not XRN2, is specifically regulated during dark adaptation

Since we verified that XRN2 and PAPD4 accumulate mainly in retinal neurons, we studied the functional regulation of these genes in response to dark-adaptation. For this purpose, four groups of rats were submitted to different treatments: control, 3 hours and 24 hours of dark-adaptation, and 24 hours of dark adaptation followed by 24 hours in 12∶12 light/dark cycle. The rationale for these treatments was to investigate the fast and slow responses to dark adaptation, and also possible long-lasting changes in expression, which could persist independently of the ambient light levels.

As shown in Figure 9, our results indicated that gene expression levels of XRN2 and PAPD4 in the retina were not significantly affected by 3 and 24 hours of dark adaptation, neither for 24 hours of dark adaptation followed by 24 hours of normal light/dark cycle (n = 6).

**Figure 11:**
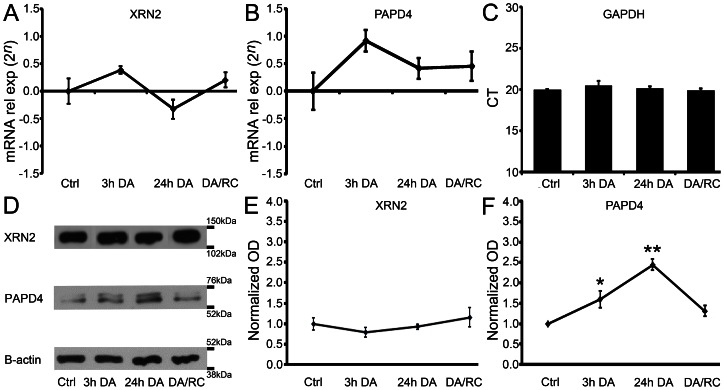
PAPD4, but not XRN2, is regulated by ambient light levels. (A–C) Using quantitative real time PCR, we compared XRN2 and PAPD4 gene expression levels after 3 and 24 hours of dark-adaptation (3h DA and 24h DA, respectively) and 24 hours of dark-adaptation followed by return to 12∶12 light/dark cycle (DA/RC) for 24 hours (n = 6). We were not able to detect changes in XRN2 and PAPD4 gene expression levels in any experimental condition. In these experiments, GAPDH abundance was used as internal control. (D–F) Interestingly, we observed upregulation in PAPD4 protein levels after 3 hours (160%, *P*<0.05) and 24 hours (244%, *P*<0.01) of dark-adaptation when compared to controls. No significant changes were observed in XRN2 protein levels. In these experiments, beta-actin abundance was used as internal control (n = 4). *P<0.05, **P<0.01 *vs.* control in Tukey's pairwise comparisons after one-way ANOVA.

Although gene expression levels of XRN2 and PAPD4 in the retina were not altered by dark-adaptation, a functional regulation of protein levels could be triggered by post-transcriptional mechanisms. To aim this question, we performed western blotting experiments for these proteins in the same experimental conditions (n = 4). Our results indicated that XRN2 protein levels were not affected in any condition when compared to controls. On the other hand, PAPD4 protein levels in the retina were significantly upregulated after 3 hours (160%, *P*<0.05) and 24 hours (244%, *P*<0.01) of dark-adaptation, when compared to controls (Fig. 11). These results revealed that levels of PAPD4, but not XRN2, are governed by ambient light levels. Moreover, the combination of our results disclosed that PAPD4 is promptly, fast upregulated by dark-adaptation, in a transcription-independent way.

### PAPD4 is differentially regulated by ambient light levels in distinct neuronal populations

Since our results revealed that ambient light levels control PAPD4 accumulation in the retina, we next examined regulation of PAPD4 levels triggered by dark-adaptation in specific retinal neurons (n = 4).

**Figure 12:**
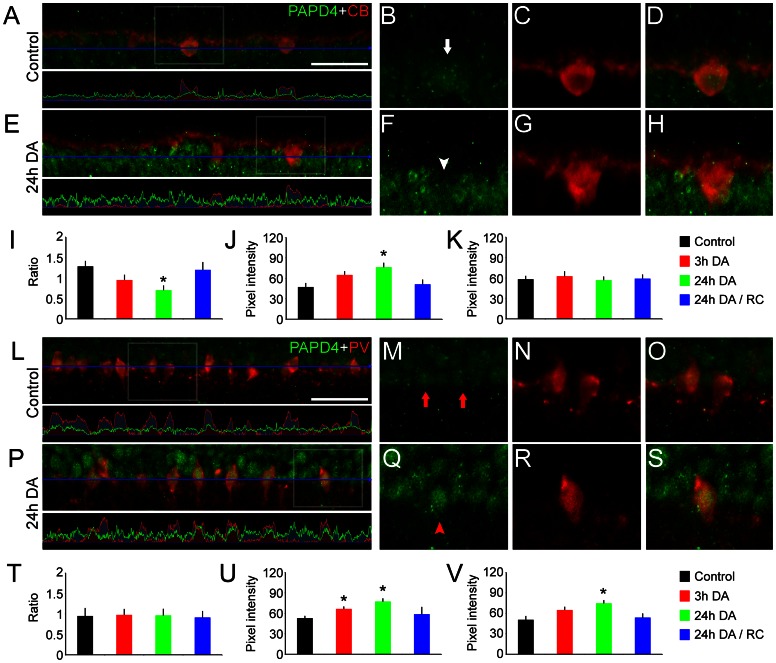
Dark-adaptation regulates PAPD4 levels in specific retinal cells. In order to quantify changes in PAPD4 driven by ambient light conditions in specific retinal neurons, we performed double-labeling experiments with different cell markers. (A, upper) As previously demonstrated in this study, using anti-calbindin (CB, red), we were able to detect PAPD4 (green) in horizontal cells. (A, lower) By analyzing the intensity pixels profile, we verified that PAPD4 green signal is regular throughout the space demarcated with the blue line. (B–D) In high magnification of selected areas, we observed PAPD4 labeling in horizontal cells (white arrow). (E, upper) In 24 hours dark-adapted retinas, it is possible to see increase in PAPD4 labeling. (E, lower) In the quantification profile, we confirmed that overall level of green channel is higher, but not in the areas defined by the red channel as horizontal cells, suggesting that PAPD4 is upregulated in other cells within the inner plexiform layer. (F–H) In high magnification of selected areas, we observed that PAPD4 labeling in horizontal cells is not especially strong in 24 hour dark-adapted retinas (white arrowhead). (I) Analysis of the ratio between the mean pixel intensity within horizontal cells and the mean pixel intensity of the overall signal detected in the same line. Our results indicated that, when compared to controls (1.28±0.12), PAPD4 labeling ratio changes in 24 hours dark-adapted retinas (0.70±0.11, *P*<0.05). (J) Quantification of the mean pixel intensity of the green channel in the outer margin of the inner nuclear layer, as defined by a single horizontal line. When compared to controls (47.25±5.30), PAPD4 signal is significantly higher in 24 hours dark-adapted retinas (76.25±6.02, *P*<0.05). (K) Comparison of mean pixel intensity of PAPD4 labeling in horizontal cells. In fact, we were not able to detect significant changes in PAPD4 labeling in horizontal cells. (L, upper) We were able to detect PAPD4 (green) in specific amacrine cell populations, characterized by the accumulation of parvalbumin (PV, red). (L, lower) As indicated by the intensity pixels profile, we verified that the green signal is regular throughout the inner border of the inner nuclear layer. (M–O) In high magnification of selected areas, we were able to detect PAPD4 in amacrine cells (red arrows). (P, upper) PAPD4 and PV double-labeling experiments were also performed in 24 dark-adapted retinas. (P, lower) Intensity profile indicated a regular distribution of PAPD4 in the inner border of the inner nuclear layer. (Q–S) In high magnification of selected areas it is possible to observe steady, uniform PAPD4 labeling in this region. (T) Analysis of the ratio between the mean pixel intensity within amacrine cells and the mean pixel intensity of the overall signal detected in the same line revealed no evident changes in PAPD4 distribution. (U) We observed an overall increase in PAPD4 labeling, when comparing controls (53.01±2.86) to 3 (66.52±3.12, *P*<0.05) and 24 (77.25±4.29, *P*<0.05) hours dark-adapted retinas. (V) PAPD4 intensity labeling in PV-positive amacrine cells was also higher, when comparing controls (50.65±4.93) to 24-dark adapted retinas (74.66±4.23, *P*<0.05). *P<0.05 *vs.* control in Tukey's pairwise comparisons after one-way ANOVA (n = 4). Scale bar: 60 µm.

In retinas from dark-reared animals, PAPD4 labeling was faint in horizontal cells, considering the intensity observed in surrounding cells located at the same stratum in the inner nuclear layer. In other words, we verified an overall increase in PAPD4 labeling, but levels in horizontal cells remained unchanged. Accordingly, the ratio between mean pixel intensity in horizontal cells in relation to overall mean pixel intensity at the same stratum differed when comparing controls (1.28±0.12) to 24 hours dark-adapted retinas (0.70±0.11, *P*<0.05). We also performed quantification of the mean pixel intensity in the outer margin of the inner nuclear layer. When compared to controls (47.25±5.30), PAPD4 signal is significantly higher in 24 hours dark-adapted retinas (76.25±6.02, *P*<0.05). However, as we mentioned, we were not able to detect remarkable changes in labeling intensity of PAPD4 in horizontal cells after 3 and 24 hours of dark-adaptation, neither in 24 hours after the return to the 12∶12 light /dark cycle (Fig. 12).

We also examined the regulation of PAPD4 in parvalbumin-positive cells, a marker of specific amacrine cell populations located in the inner border of the inner nuclear layer. We were able to detect an overall increase in PAPD4 signal in this stratum, when comparing controls (53.01±2.86) to 3 (66.52±3.12, *P*<0.05) and 24 (77.25±4.29, *P*<0.05) hours dark-adapted retinas (Fig. 12). Furthermore, our results confirmed that PAPD4 labeling is significantly upregulated by dark-adaptation in these amacrine cells, when comparing values from controls (50.65±4.93) to 24-dark adapted retinas (74.66±4.23, *P*<0.05).

## Discussion

In the nervous system, control of gene expression by miRNAs has been investigated in several, distinct, physiological processes [31], [32]. Recent studies indicated that the action of these short nucleotide sequences on specific mRNAs seems to follow stochastic, rather than deterministic, regime [33]. Therefore, width of specific miRNA activity on mRNA targets also depends on intracellular concentrations, which obviously relies on the balance of the synthesis and degradation processes [16].

In this study, we reported the expression of newly investigated genes XRN2 and PAPD4 in the retina. However, although recently described, these genes have been associated to essential processes in miRNA stability [16]. In a series of experiments, XRN2 was conclusively related to the extinction of miRNA activity rather than to the simple clearance of inactive miRNAs [34].On the other hand, PAPD4 was described as a regulatory cytoplasmic poly(A) polymerase, being responsible for the 3′-terminal adenylation of miRNA after unwinding of the duplex. This process, as a subsequent step next to Dicer processing, plays an important role in selective miRNA stability [15]. Considering the roles of miRNAs in development and cell differentiation [1], [2], [3], [4], [5], it was somewhat surprising that these genes were not obviously expressed by neuronal progenitors. Indeed, it has been proposed that regulation of specific miRNA stability and degradation provides a precise control of the cell physiology [35].

Control of gene expression in neuronal function is finely and specifically adjusted depending on the physiological process [36]. For example, adaptation to ambient light levels evolves intricate regulation to the establishment of adequate processing of visual information [37], [38]. It has been proposed that this fine tuning of the visual system should take place in the initial processing layers, as indicated by mathematical/computational modeling [39], [40], psychophysical findings [41] and cellular/molecular evidences [42].

Herein, we demonstrated that dark-adaptation regulates PAPD4, but not XRN2, expression in the retina. These results reveal changes in the balance of miRNA stability and degradation in response to ambient light levels. Moreover, PAPD4 changes were verified in specific retinal neurons, such as amacrine cells, which have important circuitry-related roles, including tuning of the general activity in retinal pathways [43]. After all, it was not surprising that regulation of miRNA activity should take place in this cell type in response to dark-adaptation.

Finally, it should be stressed that although the role of specific miRNAs in response to light levels is an interesting question, which obviously requires further and careful investigations, we were able to demonstrate that miRNA-stability related proteins are expressed preferentially by neurons, and are regulated by ambient demands. Considering the role of these proteins, it is appealing that they should provide an additional control mechanism of miRNA-related activities in the nervous system.
